# Single-nucleotide polymorphisms within the BK polyomavirus non-coding control region are genotype-associated

**DOI:** 10.1128/spectrum.00494-25

**Published:** 2025-06-24

**Authors:** Tiana A. Walder, Elizabeth A. Odegard, Heidi L. Meeds, Steven B. Kleiboeker, Assem Ziady, Anthony Sabulski, Sonata Jodele, Alix E. Seif, Stella M. Davies, Benjamin L. Laskin, Jason T. Blackard

**Affiliations:** 1 Division of Digestive Diseases, University of Cincinnati College of Medicine 12303, Cincinnati, Ohio, USA; 2 Eurofins Viracor Laboratories, Lenexa, Kansas, USA; 3 Department of Pediatrics, University of Cincinnati College of Medicine 12303, Cincinnati, Ohio, USA; 4 Division of Bone Marrow Transplantation and Immune Deficiency, Cincinnati Children’s Hospital Medical Center, Cincinnati, Ohio, USA; 5 Perelman School of Medicine, University of Pennsylvania 6572, Philadelphia, Pennsylvania, USA; 6 Division of Oncology, Children’s Hospital of Philadelphia, Philadelphia, Pennsylvania, USA; 7 Division of Nephrology, Children’s Hospital of Philadelphia, Philadelphia, Pennsylvania, USA; Penn State College of Medicine, Hershey, Pennsylvania, USA

**Keywords:** BK polyomavirus, non-coding control region, genotype, subtype, single-nucleotide polymorphisms, viral diversity, transcription factor binding sites, hematopoietic cell transplantation

## Abstract

BK polyomavirus (BKPyV) is a non-enveloped, double-stranded, circular DNA virus that is a member of the *Polyomaviridae* family. The BKPyV genome is divided into three regions, including the non-coding control region (NCCR), the early region, and the late region. BKPyV has one of the highest mutation rates among DNA viruses, and four genotypes have been identified based on amino acid variation within the VP1 region. Mutations within the NCCR have been noted, and this region exhibits hypervariability. Here, we show that many of the point mutations observed within the NCCR are genotype-associated, termed genotype-associated polymorphisms (GAPs). These GAPs correlate with regions of hypervariability, are inherent to their genotype, and can be used to genotype clinical strains, separate from other genomic regions. We also show that these GAPs fall within predicted transcription factor binding sites and therefore provide targets for further functional studies.

**IMPORTANCE:**

BK Polyomavirus (BKPyV) is the cause of hemorrhagic cystitis in hematopoietic cell transplant recipients and BKPyV-associated nephropathy in renal transplant recipients and thus is an important determinant of transplant outcome. The viral mechanisms leading to disease manifestation remain to be thoroughly explored, but viral genetic variation has emerged as an area of interest. Understanding genomic diversity between and within BKPyV genotypes can provide sites of interest that may ultimately improve screening strategies and provide insights into the viral factors that contribute to disease.

## INTRODUCTION

BK polyomavirus (BKPyV) is a member of the *Polyomaviridae* family and is one of 14 human polyomaviruses reported to date (1). The virus was first isolated from the urine of an immunocompromised patient in 1971 (2). BKPyV is an opportunistic pathogen and has a seroprevalence of 80–90% globally (3–6). While primary infection occurs commonly before the age of 10, the virus remains in an inactive state in the epithelium of the kidneys, ureters, and bladder, and infection is life-long (7, 8). Immunosuppression (usually following transplantation) can lead to viral reactivation and uncontrolled viral replication resulting in BKPyV-associated diseases. Up to 25% of hematopoietic stem cell transplant (HSCT) recipients develop hemorrhagic cystitis, and 1–5% of kidney transplant recipients develop BKPyV-associated nephropathy, leading to an increased risk of allograft dysfunction (9, 10). These clinical manifestations make the virus an important consideration for transplantation; however, the mechanisms by which viral reactivation leads to disease remain poorly understood.

The BKPyV virion consists of a non-enveloped icosahedral capsid containing the ~5 kilobase genome packaged around host-derived cellular histones, generating a mini chromosome (11). The BKPyV genome is divided into three regions—the non-coding control region (NCCR), the early coding region, and the late coding region (12). The early coding region contains genes that encode for the large and small tumor antigens (TAg and tAg) and the truncated TAg. The former two have roles in modulating the host cell cycle to regulate viral replication, while the function of the truncated TAg is not well known (13, 14). The late coding region contains genes that encode for the three structural proteins—VP1, VP2, and VP3—as well as the agnoprotein which plays a role in virion assembly, maturation, and/or release (12). The NCCR is the site of transcriptional control for BKPyV. In an archetype or wild-type BKPyV strain, the NCCR consists of 376 base pairs (bp) divided into five blocks—O (143 bp), P (68 bp), Q (39 bp), R (63 bp), and S (63 bp) (12, 15). The O block contains the origin of replication; however, multiple transcription factor binding sites (TFBSs) are present across the entire NCCR, including those for Sp1, NF1, NF-kB, and Ets-1, and are known to regulate transcription of BKPyV early and late genes (16–18).

BKPyV genomic diversity is of particular interest in the field. There are four BKPyV genotypes (I–IV), and genotypes I and IV are further divided into subtypes (19–21). Globally, genotype I is the most prevalent, with genotypes II and III being rarely found (22). Historically, BKPyV genotype has been classified based on variability within protein-coding regions of the genome, specifically the VP1 region. In 1993, Jin et al. described a 327 bp region termed the typing region that was used to distinguish BKPyV genotypes (20, 21). More recently, another group reported that a 100 bp region within the VP1 was sufficient for determining BKPyV genotype/subtype (22). Importantly, studies have shown that BKPyV genetic diversity impacts characteristics such as cell tropism, replication rates, and pathogenicity (22–24).

The NCCR represents a hotspot for variation within the BKPyV genome (12). Rearrangements are a common occurrence and involve the duplication or deletion of the P, Q, R, and/or S blocks. Rearrangements are typically associated with disease, and strains with rearranged NCCRs exhibit increased early gene expression but decreased late gene expression when compared to archetype strains, likely due to changes in TFBSs (12, 15, 16, 25, 26). Aside from rearrangements, NCCR variability has also been described through the presence of single-nucleotide polymorphisms (SNPs) (17, 27, 28). These SNPs are naturally occurring and may reside within TFBSs that influence viral replication. For instance, one group showed that a point mutation(s) within aSp1 binding site impedes binding and can alter viral gene expression, thus allowing the viral strain to mimic characteristics of a BKPyV strain with a rearranged NCCR (15, 16). Similarly, interfering with the NF-1 binding site also alters viral gene expression. Importantly, there are several other proven and putative TFBSs within the BKPyV NCCR; however, the effects of point mutations within their binding sites have not been thoroughly explored.

BKPyV reactivation leading to disease in immunocompromised patients is an ever-present issue in the transplant community, and the role that viral diversity plays in disease manifestation and pathogenesis remains unclear. Transcriptional control of viral genes and its contribution to BKPyV-associated diseases is of particular interest. Thus, we performed bioinformatic analysis of viral sequences extracted from the urine samples of HSCT recipients and GenBank reference sequences to identify regions of variability within the BKPyV NCCR to serve as candidates for further examination to determine the contribution of genetic diversity to disease.

## MATERIALS AND METHODS

### Selection of references

BKPyV references were extracted from GenBank for initial analysis of NCCR polymorphisms as well as genotype determination of patient sample sequences. Five references were selected for each of the seven major BKPyV groups (Ia, Ib1, Ib2, Ic, II, III, and IV) such that each reference had a different country of origin to maximize the geographic variation among BKPyV strains.

### Patient samples

Urine samples from a previously established cohort of 166 HSCT recipients at Cincinnati Children’s Hospital Medical Center and Children’s Hospital of Philadelphia were included (9, 29). Overall, 160 samples were collected 1 month post-transplantation, while six samples were collected 12 months post-transplantation. All samples were positive for BKPyV DNA by quantitative polymerase chain reaction performed at Viracor-Eurofins with a lower limit of detection of 500 copies/mL. The study was approved by institutional review boards at both institutions, and written informed consent was provided by all subjects.

### PCR amplification

A method previously developed by our group was utilized to process urine samples and amplify BKPyV DNA present in the samples (30). Briefly, viral DNA was extracted from 1 mL urine samples using the QIAamp UltraSens Virus Kit according to the manufacturer’s instructions with minor adjustments to centrifuge spin speeds (i.e., the initial spin was run at 4,000 × *g* for 4 minutes, and the lysate spin was run at 3,000 × *g* for 1 minute). Rolling circle amplification was then performed using Cytiva Life Sciences’ TempliPhi kit. 1 μL of extracted DNA was added to 5 μL of sample buffer, and the mixture was heated to 95°C for 3 minutes. 5 μL of reaction buffer and 0.2 μL of enzyme mix were added to the mixtures. The mixtures were then incubated at 30°C for 18 hours followed by a 10 minute 65°C heat inactivation step. The DNA was linearized using BamHI (New England Biolabs) through a 1 hour incubation period at 37°C. Full-length PCR was performed using the primers BK1731F (5′—GGG GGA TCC AGA TGA AAA CCT TAG GGG CTT TAG—3′) and BK1739R (5′—GGA TCC CCC ATT TCT GGG TTT AGG AAG CAT TCT AC—3′) (31). Amplification conditions were 93°C for 3 minutes then 25 cycles of 93°C for 30 seconds, 59°C for 15 seconds, and 68°C for 3 minutes followed by 10 minutes at 72°C. PCR products were run on a 1% agarose gel and extracted using the QIAquick gel extraction kit according to the manufacturer’s instructions. Samples were sent to the University of Cincinnati College of Medicine Genomics, Epigenomics and Sequencing Core for next-generation sequencing (NGS). Library preparation was completed using the NEBNext Ultra II FS DNA Library Prep Kit and sequences on either an Illumina HiSeq 100 with the setting SR 1 × 51 bp or an Illumina NextSeq 2000 with the setting PE 2 × 61 bp.

### Data analysis

Raw NGS reads were mapped to the genotype Ia reference AB263926 (unless otherwise stated) that exhibits an archetype NCCR structure using UGENE 50.0 and the Bowtie2 method to generate consensus sequences for each sample (32). This reference has an NCCR that is more closely related to the wild-type BKPyV strain described by Martí-Carreras et al. than archetype strains of genotypes II–IV (33). All sample sequences exhibited an archetype NCCR. Multiple sequence alignments were performed in ClustalX 2.1, and interpatient genetic distances were calculated in MEGA11 using the Kimura 2-parameter model (34, 35). Additional phylogenetic inference was performed for the NCCR alignment using the BEAST family of programs v1.10.4 and a Bayesian Markov chain Monte Carlo approach (36). The HKY substitution model was used with the gamma site heterogeneity model, an uncorrelated relaxed clock with lognormal distribution, a chain length of 200,000,000, and 10,000 logged parameters. Results were visualized in Tracer v1.7.2 to confirm chain convergence and appropriate effective sampling size (ESS). ESS values were >900, indicating effective sampling. TreeAnnotator v1.10.4 was used with a burn-in of 10% to create a consensus phylogenetic tree and visualized in FigTree v1.4.4. SNPs were identified in AliView (37). SNPs were noted if at least three sequences, irrespective of genotype, exhibited the mutation. SNPs were then classified as being genotype -associated if at least 50% of the sequences within a genotype exhibited the SNP.

### Genotype determination

Patient samples were genotyped prior to SNP analysis. To determine genotype, multiple sequence alignments were performed in which pairwise comparisons were made between sample sequences and references of known BKPyV genotype. Phylogenetic trees were generated, and the genotype for each urine sample was determined based on the references with which each sample clustered most closely.

### *In silico* analysis of transcription factor binding sites

To predict the effect of genotype-associated polymorphisms (GAPs) on TFBSs, the tool PROMO was utilized (38, 39). Transcription factors were limited to human factors, and the maximum matrix dissimilarity rate was 15%.

## RESULTS

### Genotype-associated single-nucleotide polymorphisms are present in BKPyV NCCR

**Fig 1:**
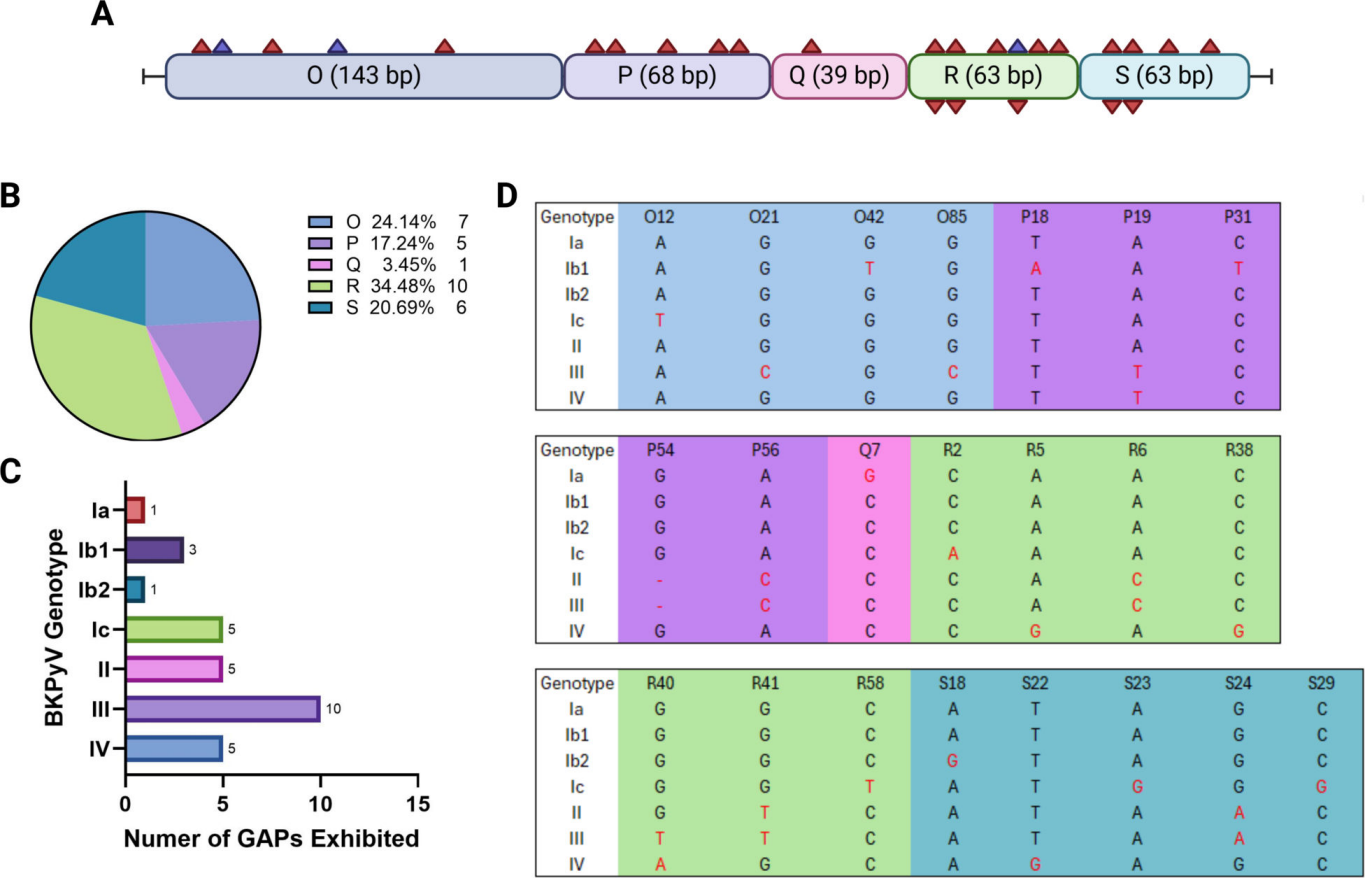
The NCCRs of 166 study sequences and 35 reference sequences were analyzed for the presence of SNPs. (A) Diagram depicting all SNPs identified with their locations in each NCCR block. Red triangles depict positions at which only one SNP was observed. Purple squares depict positions where two SNPs were observed. (B) Distribution of SNPs across NCCR blocks. (C) Number of GAPs exhibited by each BKPyV genotype. All subtypes exhibited at least 1 GAP. (D) The positions of each GAP identified and the nucleotide change that occurs when compared to other genotypes. GAPs are denoted by the red letters in each column.

A multiple sequence alignment was performed in which 35 BKPyV references (five each for genotypes Ia, Ib1, Ib2, Ic, II, III, and IV) were compared. Examination of the NCCR—encompassing nucleotides 1 to 376 of the full-length viral genome—revealed multiple SNPs. In all, 29 SNPs were observed in at least three study sequences as shown in Table 1. The distribution of SNPs among the NCCR blocks showed that 24.1% (*n* = 7) occurred in O, 17.2% (*n* = 5) in P, 3.4% (*n* = 1) in Q, 34.5% (*n* = 10) in R, and 20.7% (*n* = 6) in S (Fig. 1A and B). Two distinct SNPs were observed at three nucleotide positions—O12, O42, and R40.

**TABLE 1:** Naturally-occuring SNPs in the BKPyV NCCR^*a*^

Position in NCCR	SNP	Associated genotype
O7	A → C	N/A
O12	ΔA	N/A
O12	A → T	Ic
O21	G → C	III
O42	G → T	Ib1
O42	G → C	N/A
O85	G → C	III
P18	T → A	Ib1
P19	A → T	IV, III
P31	C → T	Ib1
P54	ΔG	II, III
P56	A → C	II, III
Q7	C--> → G	Ia
R2	C--> → A	Ic
R4	A--> → G	N/A
R5	A--> → G	IV
R6	A--> → C	II, III
R38	C--> → G	IV
R40	G--> → A	IV
R40	G--> → T	III
R41	G--> → T	II, III
R48	G--> → C	N/A
R58	C--> → T	Ic
S18	A --> → G	Ib2
S22	T--> → G	IV
S23	A --> → G	Ic
S24	G--> → A	II, III
S29	C--> → G	Ic
S50	T insrtn	N/A

^*a*^ The NCCRs of 35 BKPyV references were evaluated for the presence of SNPs. SNPs were noted if at least three sequences exhibited the same SNP. In the “Associated Ggenotype” column, “N/A” indicates that the mutation was not genotype-associated, but occurred in a notable subset of sequences. Results were validated through further examination of 166 patient sequences, in which the same mutations were present.

In all, 23 (79.3%) of the observed SNPs were present in references such that those within the same genotype exhibited the same nucleotide changes and were termed GAPs (Table 1). All seven genotypes exhibited GAPs but were most common in genotype III (*n* = 9) (Fig. 1C). Interestingly, genotype II shared all 4four of its GAPs with genotype III, while genotype IV shared 1 GAP with genotype III.

To validate these findings, BKPyV was amplified from urine samples of 166 pediatric HSCT recipients. The genotype distribution for patient sample sequences was 64.5% Ia (*n* = 107), 23.5% Ib1 (*n* = 39), 6.6% Ib2 (*n* = 11), 2.4% II (*n* = 4), 0.6% III (*n* = 1), and 2.4% IV (*n* = 4). Patient samples exhibited the same GAPs observed within the references, further supporting the genotype association of these mutations. For genotypes Ib1, Ib2, Ic, and II, every GAP was present within all study sequences of that genotype.

### Hypervariability within the archetype NCCR aligns with GAPs

**Fig 2:**
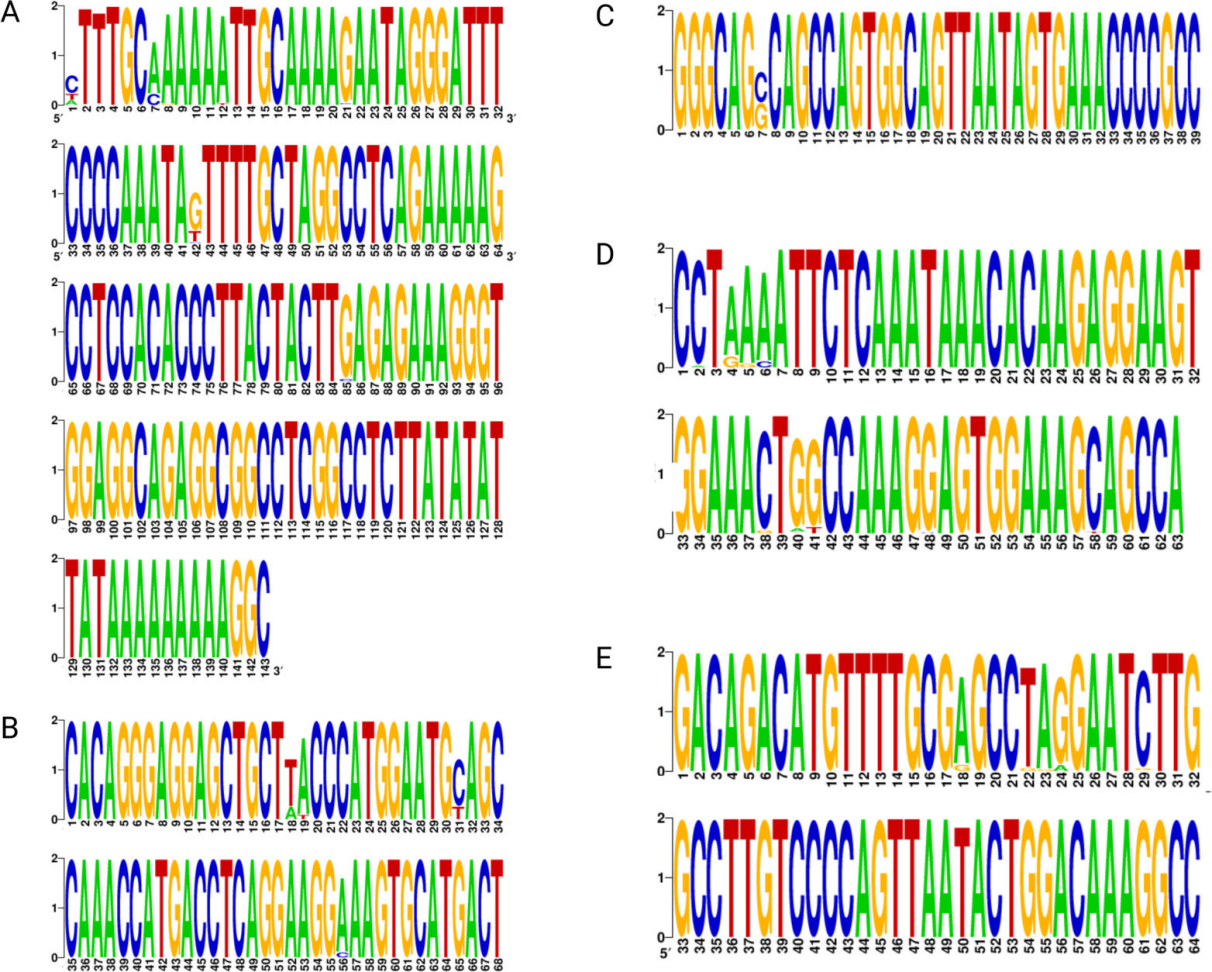
Overall, 166 study sequences and 35 references were used to generate WebLogos showing areas of variation within the BKPyV NCCR O block (**A**), *P* block (**B**), Q block (**C**), R block (**D**), and S block (**E**).

Having established that there were GAPs within the NCCR, we investigated the correlation between sites of hypervariability within the NCCR and our described GAPs. WebLogos for each block of the NCCR were generated using sample and reference sequences. Many areas of hypervariability coincided with the positions at which GAPs occurred (Fig. 2).

### Mapping to references of different genotypes does not alter GAPs

**Fig 3:**
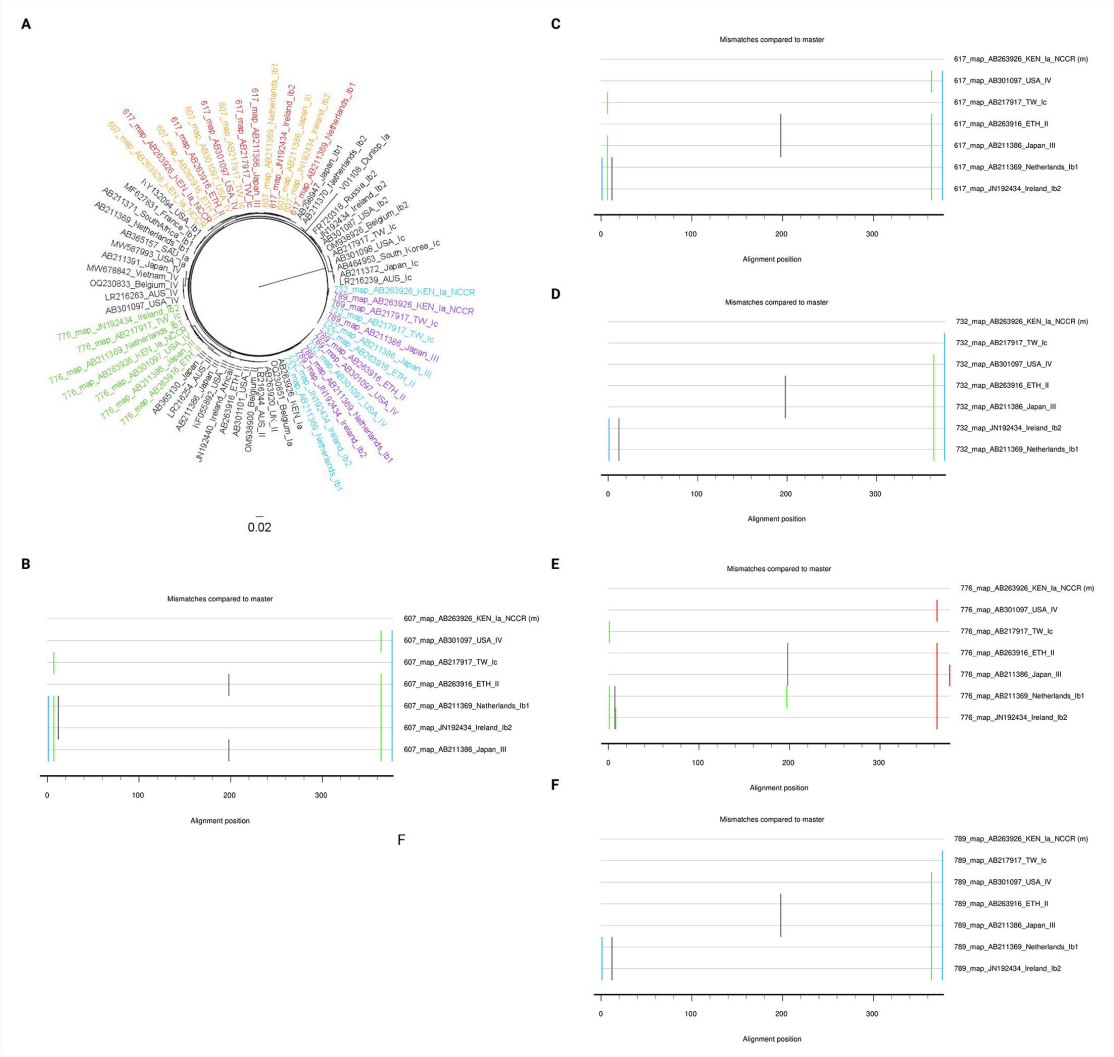
Five patient sample BKPyV sequences were used to examine the validity of the described GAPs. (A) Phylogenetic tree when full-length patient sample sequences are mapped to references of different genotypes. All samples clustered with their respective genotypes regardless of the reference utilized to generate the consensus sequence. References are labeled with their GenBank accession number, country of origin, and genotype. The sample sequences are labeled using a unique identifier and are color-coded according to the sample they were extracted from; sequences for sample 607 are highlighted in yellow, 617 in red, 732 in blue, 776 in green, and 789 in purple. (B–F) Highlighter plots depicting nucleotide differences in the NCCRs of the five patient sample sequences mapped to references of different genotypes.

We sought to verify that the observed GAPs were inherent to the genotype and not due to the methods used to generate consensus sequences. Multiple full-length consensus sequences were generated for five representative patient sample sequences—selected randomly from the patient population—by mapping NGS reads to references that represent different genotypes. The five sample sequences represented genotypes Ia, Ib1, and III. References of seven different BKPyV genotypes (Ia, Ib1, Ib2, Ic, II, III, and IV) were used to map NGS reads and generate consensus sequences. An alignment was performed using the consensus sequences generated for each sample, along with references for each genotype. A phylogenetic tree was generated, and visualization of the tree showed that—independent of the genotype to which NGS reads were mapped—the sample sequences still clustered with their respective genotypes (Fig. 3). However, phylogenetic analysis revealed some variation among sequences; thus, genetic distance was calculated for the NCCR portion of the sequences. The average genetic distance between NCCRs ranged from 0.22% to 0.54%, representing an average number of nucleotide changes from 0.64 to 2.02 (Table 2). Importantly, these nucleotide changes did not impact any of the described GAPs. Taken together, these data suggest that each GAP is specific to its respective genotype.

**TABLE 2:** Genetic distances calculated in MEGA II using the Kimura 2two-parameter model^*a*^

Sample	Genotype	Average genetic distance between consensus sequences (%)	Average number of nucleotide changes
607	Ib1	0.54%	2.02
617	Ib1	0.54%	2.02
732	Ia	0.36%	1.35
776	III	0.22%	0.84
789	Ia	0.36%	1.35

^*a*^ The average genetic distances shown are for NCCR consensus sequences of NGS reads mapped to references of genotype Ib1, Ib2, Ic, II, III, or IV compared to the consensus sequence generated for reads mapped to the genotype Ia reference.

### Archetype NCCR is sufficient for genotyping

**Fig 4:**
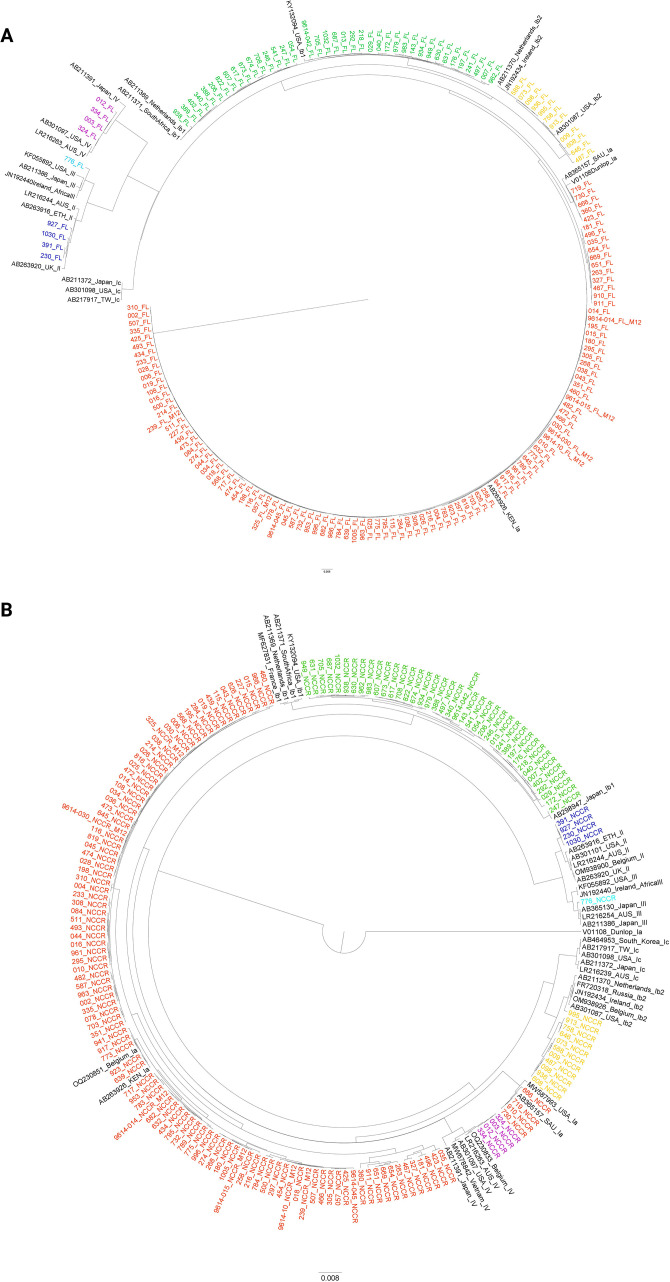
(A) Phylogenetic tree of full-length sequences excluding the NCCR of 166 study sequences and 21 reference sequences of known BKPyV genotype. (B) Phylogenetic tree of the NCCR from the same patient sample sequences seen in panel A and 35 reference sequences. For both trees, references are labeled with their GenBank accession number, country of origin, and genotype. Patient sample sequences are labeled using a unique identifier. Patient sample sequences are also color coded according to their genotype: red for Ia, yellow for Ib2, green for Ib1, dark blue for II, light blue for III, and purple for IV.

To determine if the NCCR alone was sufficient for BKPyV genotype determination, two phylogenetic trees were generated utilizing the 166 study sequences and references. The first included the full-length BKPyV genome exclusive of the NCCR (Fig. 4A), and the second included the NCCR only (Fig. 4B). The genotypes were concordant for all samples, suggesting that genotyping based on the NCCR only is satisfactory.

### Transcription factor binding sites potentially affected by GAPs

The NCCR is the site of transcriptional control, and several GAPs are positioned within TFBSs. PROMO was utilized to predict which TFBSs were affected by the presence or absence of GAPs (Table 3). Except for 085 G → C and R58 C → T, all GAPs were predicted to alter TFBSs either by inactivating an already existing binding site or introducing a new binding site. Binding sites for several transcription factors associated with BKPyV transcriptional control were predicted to be impacted, including those for NF-1, YY1, AP-2, LF-A1, PR, and GR. A previous study demonstrated that point mutations within the Sp1 site can greatly alter viral gene expression (12, 13). We have presented several GAPs that are predicted to affect many TFBSs and should be evaluated in future studies to determine their impact on BKPyV gene expression.

**TABLE 3:** Transcription factor binding sites predicted by PROMO to be affected by genotype-associated polymorphisms

Position in NCCR	GAP	Associated genotype	Potentially affected TFBS
O12	A--> → T	Ic	MBF1, NF-A, POU2F2, POU2F2B, POU2F1, CUTL1
O21	G--> → C	III	SRY, GR-alpha, GR-beta
O42	G--> → T	Ib1	HNF-3alpha, MBF1
O85	G -->→ C	III	–^*a*^
P18	T--> → A	Ib1	AP-3, FOXO3a
P19	A--> → T	IV, III	STAT5A, FOXO3a
P31	C--> → T	Ib1	GCMa
P54	ΔG	II, III	C/EBPalpha, C/EBPbeta, R2, STAT5A, c-Ets-2, p300
P56	A--> → C	II, III	C/EBPalpha, C/EBPbeta, R2, STAT5A, c-Ets-2, p300
Q7	C--> → G	Ia	AP-2alphaA
R2	C--> → A	Ic	YY1, LCR-F1, POU2F2C, Sp3, ETF
R5	A--> → G	IV	HSF1 (long), HSF1 (short), MBF1, POU3F2
R6	A--> → C	II, III	GR-alpha, MBF1, POU3F2
R38	C--> → G	IV	c-Myb, E47, IRF-3, ENKTF-1
R40	G--> → A	IV	c-Myb, E47, IRF-3, ENKTF-1
R40	G--> → T	III	C/EBPalpha, C/EBPbeta, LF-A1, E47, IRF-3
R41	G--> → T	II, III	PR, PR A, R2
R58	C--> → T	Ic	–^*a*^
S18	A --> → G	Ib2	p53, EllaE-A, E2F-1
S22	T--> → G	IV	PU.1, TTF-1, AP-2
S23	A --> → G	Ic	E47, R2, TFIIB, c-Ets-2
S24	G--> → A	II, III	FOXO3a, c-Ets-2, STAT5A, p300
S29	C--> → G	Ic	E47, R2, TFIIB, GR-alpha, c-Ets-2

^*a*^ The tool PROMO was utilized to predict transcription factor binding sites. A dash, “–”, indicates that no binding sites were predicted to be impacted by the mutation.

## DISCUSSION

Viral diversity is a major component driving the virulence of many viruses, including human immunodeficiency virus (HIV), hepatitis C virus (HCV), and SARS-CoV-2. Variability within their genomes can also influence cell tropism, replication rate, and resistance to antivirals (22). Similarly, understanding BKPyV genomic diversity is critical to understanding pathogenesis. Several studies have demonstrated functional differences between BKPyV genotypes. For example, BKPyV viral loads in the urine were higher in renal transplant recipients infected with genotype Ia relative to those infected with genotype Ib1 (24). The ability to infect target cell types was also impacted by BKPyV genotype (23). Point mutations within the viral genome may also be important. For instance, a point mutation(s) at amino acid residue 68 in the VP1 protein changes the specificity of BKPyV for one of its target receptors—GD3*—in vitro* (40).

We identified 29 SNPs, 23 of which were genotype associated. Several of these SNPs were identified previously, including O42 G → T, P18 T → A, and P31 C → T (17, 27, 28). We identified these same SNPs, both previously reported and newly discovered, as being associated with a specific genotype. We are, to our knowledge, the first to describe the mutations O7 A → C, O21 G → C, O42 G → C, P54 ΔG, R48 G → C, R58 C → T, and S24 G → A. Of these mutations, five are GAPs, and three of those five are associated with genotype II and/or III (O21 G → C, P54 ΔG, S24 G → A). Genotype II shared all of its GAPs with genotype III and could explain the close genetic relatedness between genotypes II and III. However, genotypes II and III are rare, and sequence data from these genotypes are limited. Our identification of these type II/III GAPs expands our knowledge and can increase our understanding of rare genotypes.

Several distinct approaches for determining the BKPyV genotype have been reported in the literature (19–21, 41). However, these approaches typically utilize protein-coding regions of the genome. Our analysis of the NCCR demonstrates that it is sufficient for genotyping archetype BKPyV strains. Furthermore, the GAPs described here represent an additional tool for genotyping BKPyV and can serve as targets of interest for studying the transcriptional control of viral replication. Many GAPs fall within predicted TFBSs and promoter elements. For example, the GAPs at R38, R40, and R41 all fall within the same NF-1 binding motif, Q7 falls within an AP-2 motif, and P54 and P56 fall within a CMV ie-1 promoter (17). Mutations affecting Sp1 transcription factor binding were shown previously to regulate bidirectional expression of early and late viral genes, miRNA levels, and replication rates (15, 16, 42). Other transcription factors are also implicated in the transcription of BKPyV genes. The activities of NF-1 and NF-κB control both early and late gene expression (18, 43–46). NF-AT and p53 repress early BKPyV gene expression, while AP-2 has been implicated in the control of related simian polyomavirus, SV40, through its interaction with Sp1 (47–49). NF-AT, p53, and AP-2 were also shown to have higher expression during active BKPyV infection when compared to latent infection and uninfected controls (50). As with Sp1 sites, mutations within other TFBSs may impact their ability to regulate viral gene expression and virus replication. To gain a better insight into the transcriptional control of BKPyV and possible differential regulation of distinct genotypes, additional *in vitro* investigations are required.

In conclusion, we defined GAPs within the BKPyV NCCR that are inherent to each genotype. These GAPs suggest conservation between genotypes and can be used to genotype archetype BKPyV strains through phylogenetic analysis. We also present these GAPs as potential targets for future examination to better understand the transcriptional control of BKPyV genes. This opportunistic pathogen is associated with hemorrhagic cystitis and nephropathy following transplantation, and understanding the mechanisms that regulate gene expression may allow for the future development of novel therapeutic strategies.

## Data Availability

Raw sequence data are available under BioProjects PRJNA1198305 and PRJNA670723. The consensus BKPyV genome sequences are available under GenBank accession numbers PQ859676–PQ859841.
